# Spatiotemporal variations of agricultural water footprint and its economic benefits in Xinjiang, northwestern China

**DOI:** 10.1038/s41598-021-03240-9

**Published:** 2021-12-13

**Authors:** Yinbo Li, Mingjiang Deng

**Affiliations:** 1 College of Resource and Environmental Science, Xinjiang University, Ürümqi, 830046 Xinjiang People’s Republic of China; 2 General College Key Laboratory of Smart City and Environmental Modeling, Xinjiang University, Ürümqi, 830046 Xinjiang People’s Republic of China

**Keywords:** Climate change, Hydrology

## Abstract

Agriculture is the largest water user and is the main driving force behind water stress in Xinjiang, northwestern China. In this study, the water footprint (WF) (blue, green and gray WF) of main crop production and their temporal and spatial characteristics in Xinjiang were estimated in 2006, 2010, 2014 and 2018. The blue water footprint deficit (BWF_d_) was conducted and food productivity and economic benefits of WF were also analyzed via the water consumption per output value (food productivity and economic benefits). The results reveal that the WF increased from 22.75 to 44.16 billion m^3^ during 2006–2018 in Xinjiang, of which cotton, corn and wheat are main contributors of WF. In terms of different regions, corn has the largest WF in north Xinjiang and cotton has the largest WF in south and east Xinjiang. The BWF_d_ broadened from − 11.51 to + 13.26 billion m^3^ in Xinjiang with the largest increased BWF_d_ in Kashgar (from − 3.35 to 1.40 billion m^3^) and Aksu (from − 2.92 to 2.23 billion m^3^) of south Xinjiang and in Shihezi (from − 0.11 to 2.90 billion m^3^) of north Xinjiang. In addition, the water footprint food productivity does not well correspond with the water footprint economic benefits in prefectures of Xinjiang. It means we should consider the food yields priority and economic benefits priority to formulate a scientific and effective supervisor mode to realize the sustainable management of agricultural water in prefectures of Xinjiang.

## Introduction

Water shortage, an important issue of global sustainable development^1–3^, is more pronounced in the arid regions where the environment is extremely harsh and water resources are extremely scarce^4^ and large amount of water resources are used for agriculture irrigation^5–7^. For example, water for agriculture was accounting for 94.4% of total amounts in Xinjiang, northwest China (from Xinjiang Water Resources Bulletin 2016). Excessive use for irrigation water will occupy the ecological water requirement, which brings a great challenge for ecology security. Therefore, we should firstly know the agriculture water requirement and then establish the balance between the irrigation water and the ecological water requirement^8,9^.

To quantify the irrigation water requirement for crop production, the water footprint (WF) was introduced by Hoekstra^10^ and includes blue water footprint (WF_blue_), green water footprint (WF_green_) and gray water footprint (WF_gray_): WF_blue_ is irrigation water from the surface and ground water, WF_green_ is the consumed rainwater, WF_gray_ is the volume of freshwater used to assimilate the load of pollutants based on existing ambient water quality standards^11^. This methodology has later been extensively applied to the global- and national-scale calculation for water footprint of multiple crops^6–15^. For example, Chapagain et al.^12^ calculated the global consumption water for cotton is 256 Gm^3^ each year during 1997–2001, of which blue, green and gray is 42%, 39% and 19%, respectively. Zhou et al.^13^ estimated the China’s averaged WF per capital and found the crop consumption reduced by 23% from 625 m^3^/cap in 1978 to 481 m^3^/cap in 2008. Among crops, more than half of the total WF_blue_ within China was from rice (51%), followed by wheat (28%). Rice (32%) and wheat (20%) together also shared half of the total WF_green_. Liu et al.^6^ assessed the total food consumption grows 35.4% from 147.0 to 162.9 million in Northwest China during 2000–2016. However, the WF related to food consumption only increased from 153.8 Gm^3^ to 159.6 Gm^3^ due to the improvement of water saving efficiency.

Researches of crops water footprint requirement (WF_r_) in Xinjiang has increased in recent decades^5,16–18^. Shen et al.^16^ calculated the WF_r_ in Tarim Basin and Junggar Basin and found it had a rapidly increasing trend with the highest value 172.2 × 10^8^ m^3^ in 2010. Wang et al.^17^ pointed out the WF_r_ of Tarim Basin was 359 × 10^8^ m^3^ in 2010 and 472 × 10^8^ m^3^ in 2015. Li et al.^5^ found the total WF_r_ of Xinjiang was 389.9 × 10^8^ m^3^ in 2018. Zhang et al.^18^ proposed that the WF_r_ increased from 73.91 × 10^8^ m^3^ in 1988 to 270.50 × 10^8^ m^3^ in 2015. We can find that the WF_r_ calculated by different groups in the same year are inconsistent because of the selected different crops and parameters (e.g., irrigation coefficient). For example, Shen et al.^16^ chosen wheat, corn, cotton, oil crops and soybeans, while wheat, corn, cotton, rice and fruit trees were selected in Wang et al.^17^. The averaged irrigation coefficient in Xinjiang was used by Shen et al.^16^ and Wang et al.^17^, while Li et al.^5^ used the irrigation coefficient of different prefectures. Several obvious deficiencies are also discovered: (1) WF_gray_ is the important contributor to Xinjiang production due to the extensive use of chemical fertilizer^19^, but their data are relative scarce for Xinjiang crops; (2) few studies estimated the difference between the actual irrigation water and WF_blue_ to measure the extent of blue water scarcity in Xinjiang; (3) the water footprint per unit of yield (WY) received more attention to reveal water productivity from the perspective of food production, while few studies describe water productivity from the perspective of economic benefits via the water footprint per output value (WV).

In this study, we are planning to address the above three issues: (1) the WF_blue_, WF_green_ and WF_gray_ for main 11 crops were calculated and their temporal and spatial characteristics were investigated in 2006, 2010, 2014 and 2018; (2) the new indicator-blue water footprint deficit (BWF_d_)^3^ were used to reveal the actual situation of water shortage in Xinjiang; (3) the WF per unit of yield (WY) and WF per unit of output value (WV) were calculated to estimate water footprint food production and economic benefits in Xinjiang. Our works could enrich indicators about water footprint of crop production and also offer an optional way for agriculture saving water in Xinjiang.

## Study area

**Figure 1:**
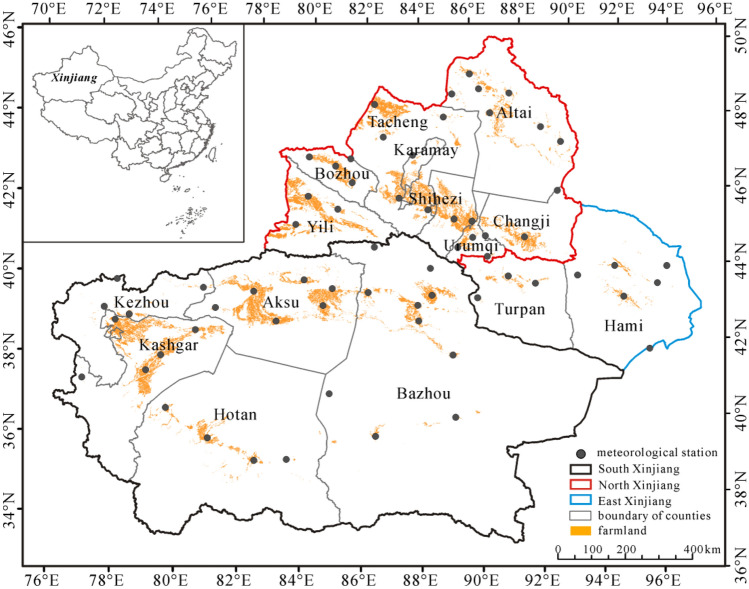
Distribution of farmlands, meteorological stations and prefectures in Xinjiang, China. Map is created in ArcGIS 10.1 and data are from National Earth System Science Data Center, National Science & Technology Infrastructure of China (http://www.geodata.cn).

Xinjiang (34°–49°N, 73°–96°E), located in the northwestern China, covers an area of approximately 166 × 10^4^ km^2^ and accounts for 1/6 of the total area in China. It has 15 prefectures including Urumqi, Changji, Shihezi, Karamay, Turpan, Hami, Altai, Tacheng, Bozhou, Yili, Aksu, Bazhou, Kezhou, Hotan and Kashgar. Based on the topography, Xinjiang is classified into three regions (north, south and east Xinjiang) (Fig. 1). North Xinjiang has eight prefectures including Urumqi, Changji, Shihezi, Karamay, Altai, Tacheng, Bozhou and Yili. South Xinjiang has five prefectures (Aksu, Bazhou, Kezhou, Hotan and Kashgar). Turpan and Hami belong to East Xinjiang.

The climate is characterized by a temperate continental climate with mean annual precipitation of < 200 mm and mean annual temperature of 9.1 °C. The oases are mainly situated in the piedmont plains and their water resources primarily result from rivers originating from precipitation and from glacial and snow melt water in the mountainous regions (i.e., Tianshan, Altai and Kunlun Mountains). The cultivated lands are distributed in the oasis regions. Water use for agricultural irrigation has been accounted for > 90% of freshwater use in Xinjiang, which is much higher than the average level of China. The planting area and yield of 11 main crops in Xinjiang were shown in Supplementary Table S1. The irrigation area of Xinjiang increased from 3856.91 × 10^3^ ha to 5573.16 × 10^3^ ha during 2006–2018 with an increase of 44.50%. Correspondingly, the crop yield increased from 3189.73 × 10^7^ kg to 4660.45 × 10^7^ kg in this interval with an increase of 46.10%. The crops planting area and their yield accounted for both > 80% in total.

## Results

### Trends of water footprint (WF)

**Figure 2:**
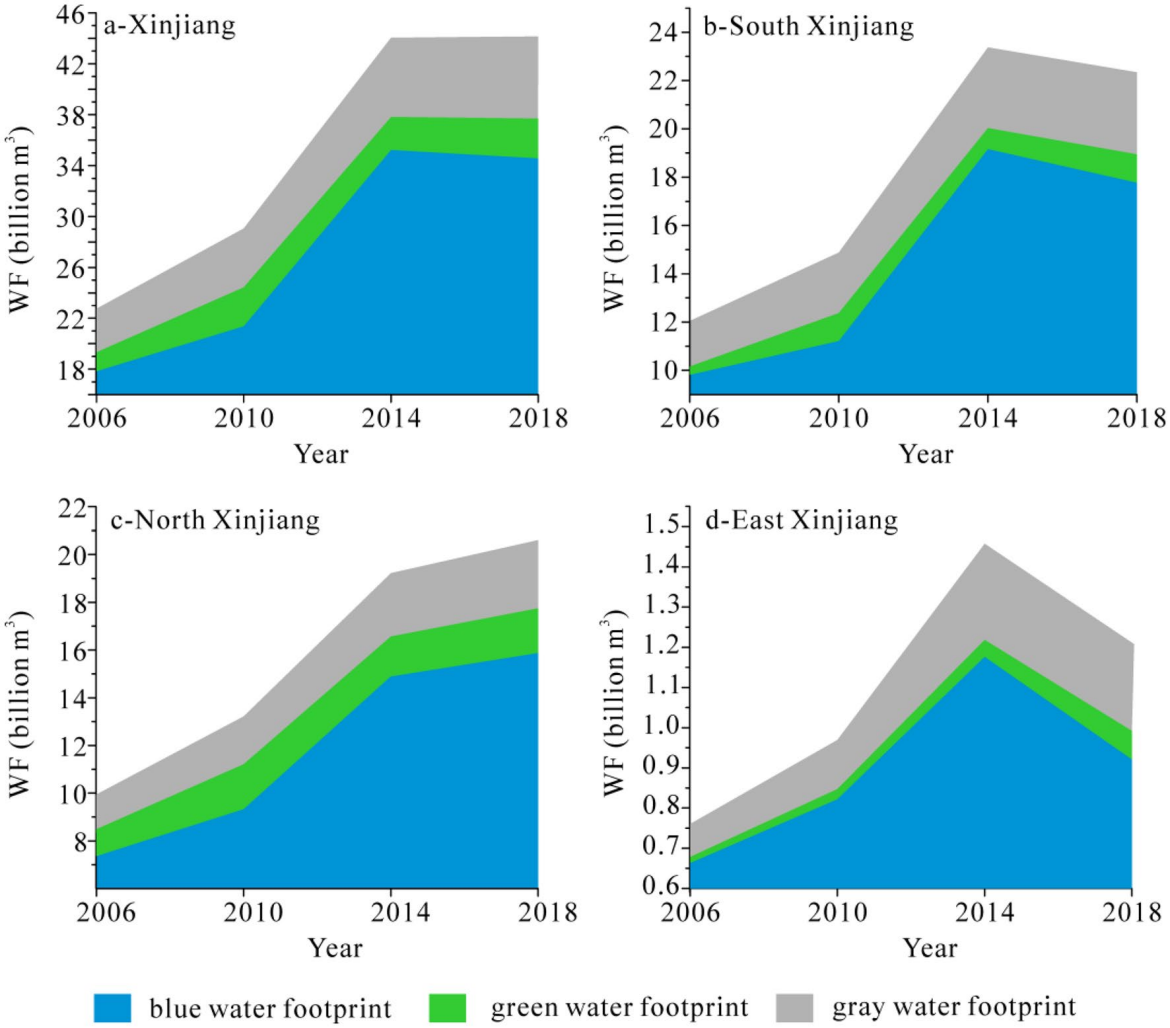
Total water footprint (WF) of crops in 2006–2018.

The WF in Xinjiang increased from 22.75 to 44.16 billion m^3^ with a rate of 2.0 billion m^3^ during 2006–2018 (Fig. 2a). The increased WF comes from the growing contributions of WF_blue_, WF_green_ and WF_gray_. In detail, it significantly increased from 17.84 to 34.58 billion m^3^ for WF_blue_, from 1.50 to 3.12 billion m^3^ for WF_green_, and from 3.41 to 6.46 billion m^3^ for WF_gray_. However, the percentages of WF_blue_, WF_green_ and WF_gray_ are basically stable in the study interval: the ratio of WF_blue_ decreased from 78.42 to 78.30%, that of WF_green_ increased from 6.61 to 7.06% and that of WF_gray_ decreased from 14.98 to 14.63%.

### Spatial features of water footprint (WF)

In terms of three regions, WF in south Xinjiang increased from 12.05 to 22.34 billion m^3^ during 2006–2018 (Fig. 2b). It increased from 9.94 to 20.61 billion m^3^ in north Xinjiang (Fig. 2c) and increased from 0.76 to 1.21 billion m^3^ in east Xinjiang (Fig. 2d). All prefectures experienced an increasing trend of WF during 2006–2018. Aksu and Kashgar had the fastest increased rates (0.38 billion m^3^ and 0.35 billion m^3^ each year), while the rates of Karamay and Urumqi were relatively in low levels (6.80 × 10^6^ m^3^ and − 6.20 × 10^5^ m^3^ each year). Among these prefectures from 2006 to 2014, the WF all increased; from 2014 to 2018, the WF in Aksu, Altay, Kezhou, Shihezi, Tacheng and Yili also increased and decreased in other regions. In 2018, the highest WF occurred in Kashgar (8.34 billion m^3^) with a contribution rate of 18.88%; the lowest WF appeared in Karamay (0.16 billion m^3^) with a contribution rate of 0.35%.

**Figure 3:**
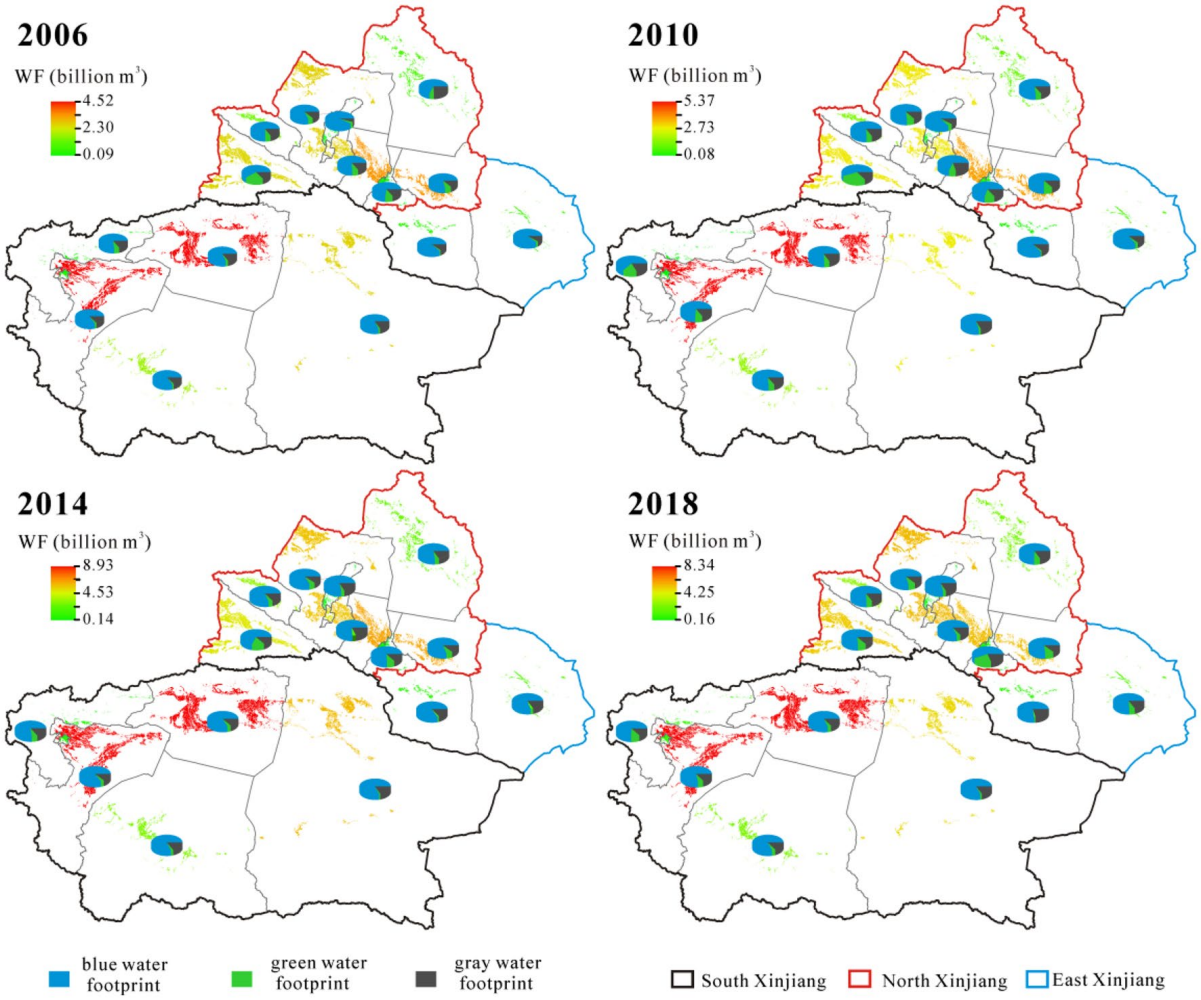
Spatial distribution of water footprint (WF) in 2006–2018. Map is created in ArcGIS 10.1.

The spatial characters of WF in 2006–2018 were shown in Fig. 3. The proportion of WF in south Xinjiang decreased from 52.95 to 50.59%, and it decreased from 3.34 to 2.73% in east Xinjiang, while it increased from 43.71 to 46.67% in north Xinjiang. In 2018, three prefectures with the highest proportion of WF in east, north and south Xinjiang were Hami (69.15%), Tacheng (23.13%) and Kashgar (37.32%), respectively.

### Water footprint (WF) of different crops

**Figure 4:**
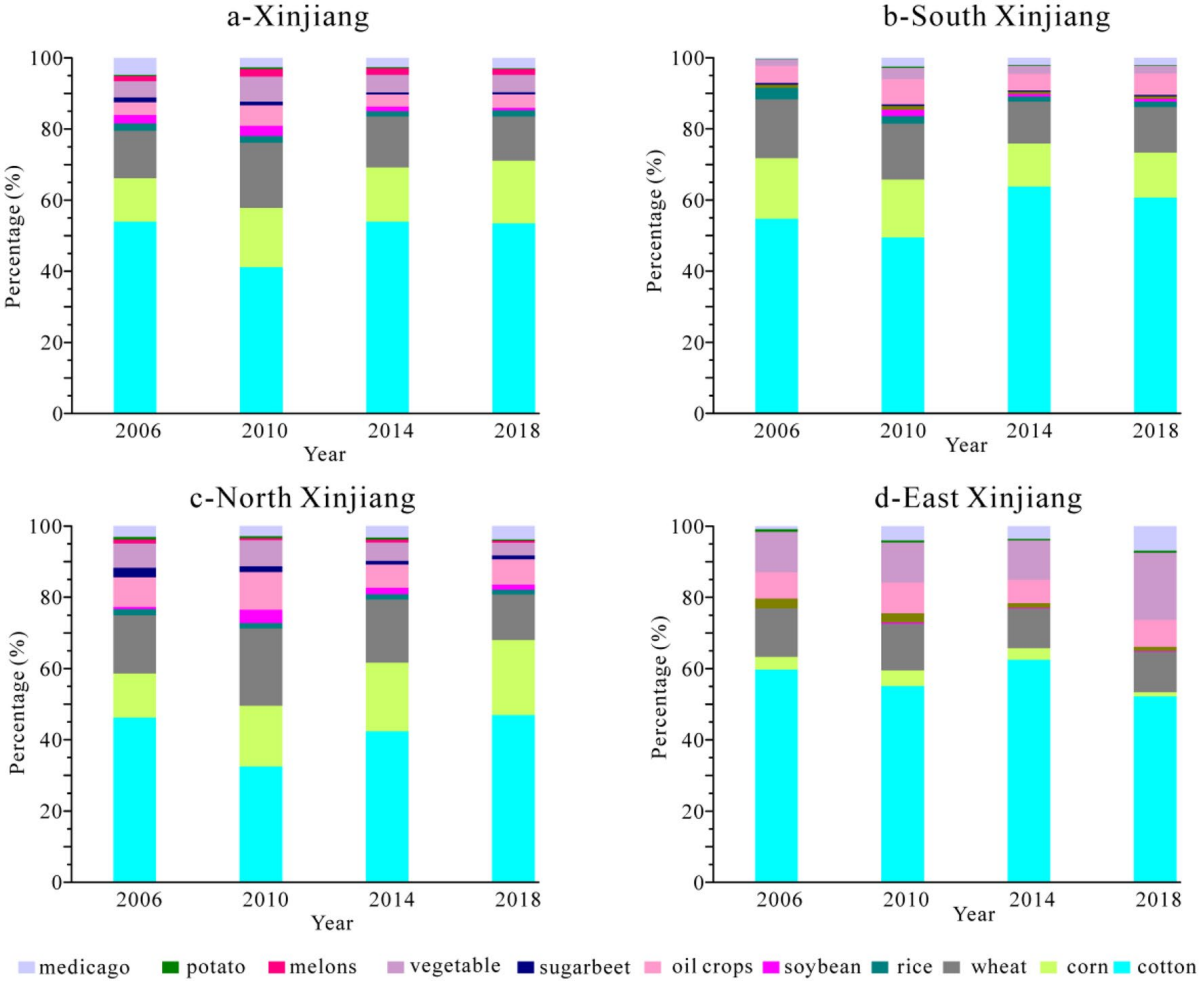
Total water footprint (WF) and contribution rate in Xinjiang during 2006–2018.

Cotton, corn and wheat are major contributors of WF in Xinjiang during 2006–2018. Their amounts increased of 55.19% from 23.14 to 35.91 billion m^3^. The WF of corn had the largest increase of 114.20% from 3.55 to 7.61 billion m^3^. The WF of rice had the smallest increase of 20.06% from 0.61 to 0.76 billion m^3^. The WF of soybeans, sugerbeet, potato, and medicago in Xinjiang decreased from 0.61 billion m^3^, 0.40 billion m^3^, 0.10 billion m^3^ and 1.38 billion m^3^ to 0.33 billion m^3^, 0.27 billion m^3^, 0.08 billion m^3^ and 1.24 billion m^3^, respectively (Fig. 4).

The contribution rates of WF were different in south Xinjiang, north Xinjiang and east Xinjiang (Fig. 4). In south and east Xinjiang, the largest contribution rate for WF among crops was cotton, accounting for 52.26–63.81%, while the smallest rate was potato, accounting for 0.10–0.72%. In north Xinjiang, the largest contribution rate was corn with an increase from 12.33 to 21%, while the contribution rates of wheat and vegetables respectively decreased from 16.33 and 6.81% to 12.79 and 3.61%. The smallest contribution rate was also potato, only accounting for 0.37–0.73%.

In terms of WF_blue_, corn is major contributor among crops and increases from 11.97 to 20.16% in north Xinjiang. Being converse with corn, the contribution rate of wheat has a decreasing trend from 14.44 to 11.08%. No obvious changes were showed in other crops. In south Xinjiang, the contribution rate of cotton increased from 55.83 to 63.02%, while that of corn and wheat decreased from 16.74 and 15.67% to 12.11 and 11.45%. In east Xinjiang, WF_blue_ of cotton, corn and wheat decreased significantly from 60.40%, 3.63%, 12.30% to 53.44%, 1.16%, 10.10%.

In terms of WF_green_, the contribution rates of cotton and corn were increased from 32.29 and 15.18% to 39.16 and 24.16%, and it was decreasing in other crops with different rates, of which the contribution rate of wheat decreased obviously from 25.33 to 15.96% in north Xinjiang. Being consistent with changes of cotton in north Xinjiang, the contribution rate of cotton increased from 46.54 to 52.17% in south Xinjiang. Corn and wheat have decreasing trends from 19.23 and 22.71% to 16.26 and 16.80%, respectively. The observed changing trends of crops in east Xinjiang are similar with that in south Xinjiang.

In terms of WF_gray_, the contribution rate of cotton was decreasing from 47.30 to 37.90%, while that of corn increased significantly from 11.86 to 23.91% in north Xinjiang. The contribution rates of other crops were basically stable. The contribution rate of cotton was increasing from 50.35 to 51.65% in south Xinjiang. In east Xinjiang, the changeable trends of the contribution rate of crops were the same as those in south Xinjiang.

Based on the above analysis, the main contributions of WF come from cotton, corn and wheat in Xinjiang during 2006–2018. The increasing contributions of WF in north Xinjiang were from WF_blue_ and WF_gray_ of corn and WF_green_ of cotton and corn, while those in south Xinjiang and east Xinjiang were from WF_blue_, WF_green_ and WF_gray_ of cotton. The contribution rate of WF_blue_ was much higher than that of WF_green_ and WF_gray_, which suggests these planting crops were mainly depended on irrigation water in Xinjiang.

### Blue water footprint deficit (BWF_d_)

During 2006–2010, the BWF_d_ increased from − 11.51 to − 6.28 billion m^3^ due to the expansion of planting areas. Although the water-saving technologies are beginning to be applied (e.g., dropper technology) in Xinjiang, this was not enough to offset the rapid increase of water demand due to the expansion of irrigation farmland since 2010. The rapid expanded irrigation farmland results in the larger BWF_d_ from − 6.28 billion m^3^ in 2010 to 13.26 billion m^3^ in 2018, leading to more severe shortage of blue water in Xinjiang.

**Figure 5:**
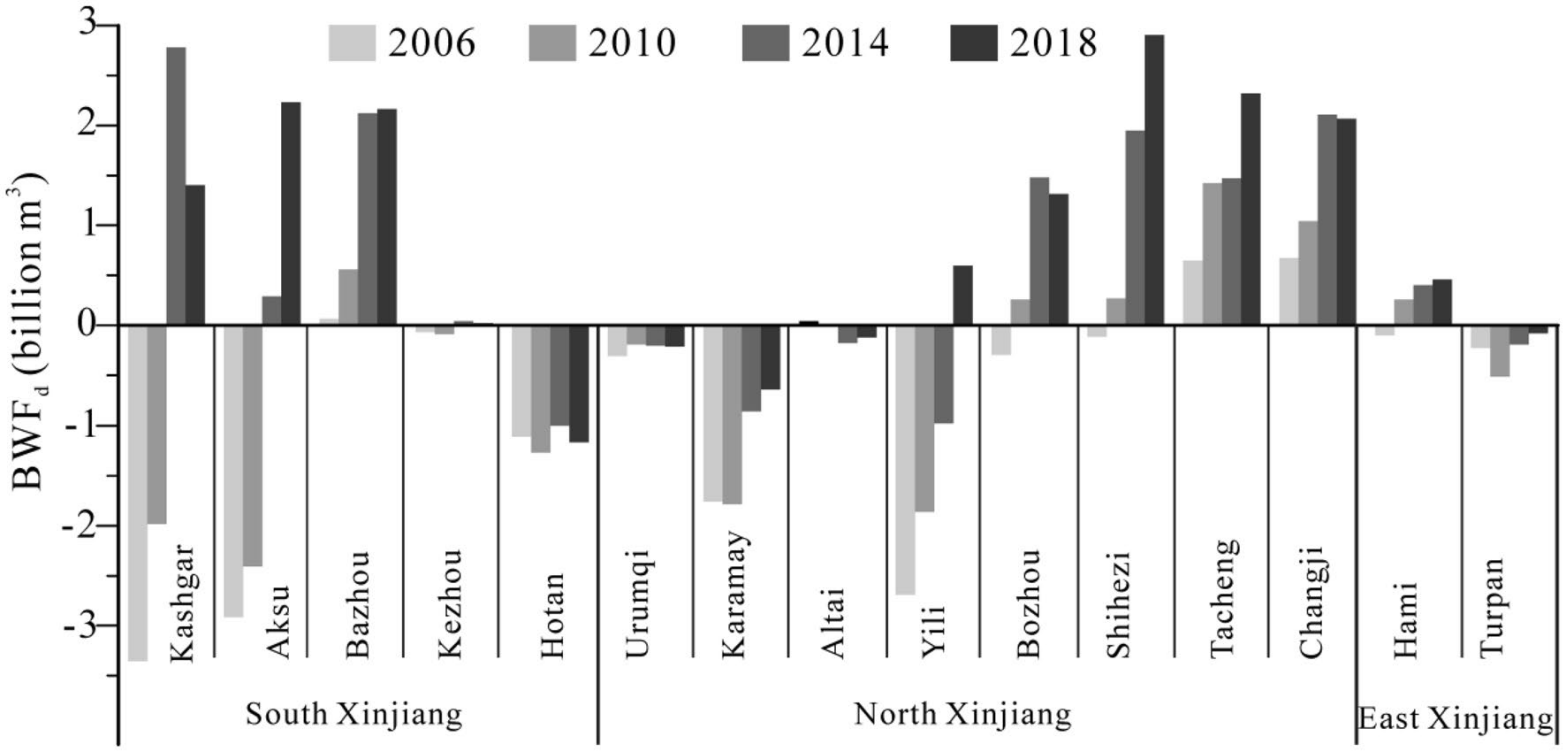
Blue water footprint deficit (BWF_d_) in prefectures of Xinjiang.

Regarding the different prefectures in three regions (Fig. 5), the BWF_d_ in south Xinjiang was lower than that in north Xinjiang. In south Xinjiang, the largest increases of BWF_d_ were showed in Kashgar (from − 3.35 billion m^3^ in 2006 to 1.40 billion m^3^ in 2018) and Aksu (from − 2.92 billion m^3^ in 2006 to 2.23 billion m^3^ in 2018). Bazhou has been in the state of BWF_d_ with an increasing trend from 0.06 billion m^3^ in 2006 to 2.16 billion m^3^ in 2018. The increased BWF_d_ was also found in Kezhou from − 0.07 billion m^3^ in 2006 to 0.02 billion m^3^ in 2018. Hotan has been in the condition of the blue water surplus during 2006–2018. In north Xinjiang, the blue water surpluses were found in Urumqi and Altai during 2006–2018. The blue water surplus was found in Yili before 2014, while the state of blue water deficit was found in Bozhou and Shihezi since 2010. Tacheng and Changji were always in the blue water deficit in the studied interval. The largest increasing BWF_d_ was showed in Tacheng from 0.65 billion m^3^ in 2006 to 2.32 billion m^3^ in 2018. In east Xinjiang, the blue water surplus was found in Turpan, while Hami was in the deficit condition of BWF_d_ during 2006–2018.

## Water footprint productivity

### Water footprint per unit of yield (WY)

**Figure 6:**
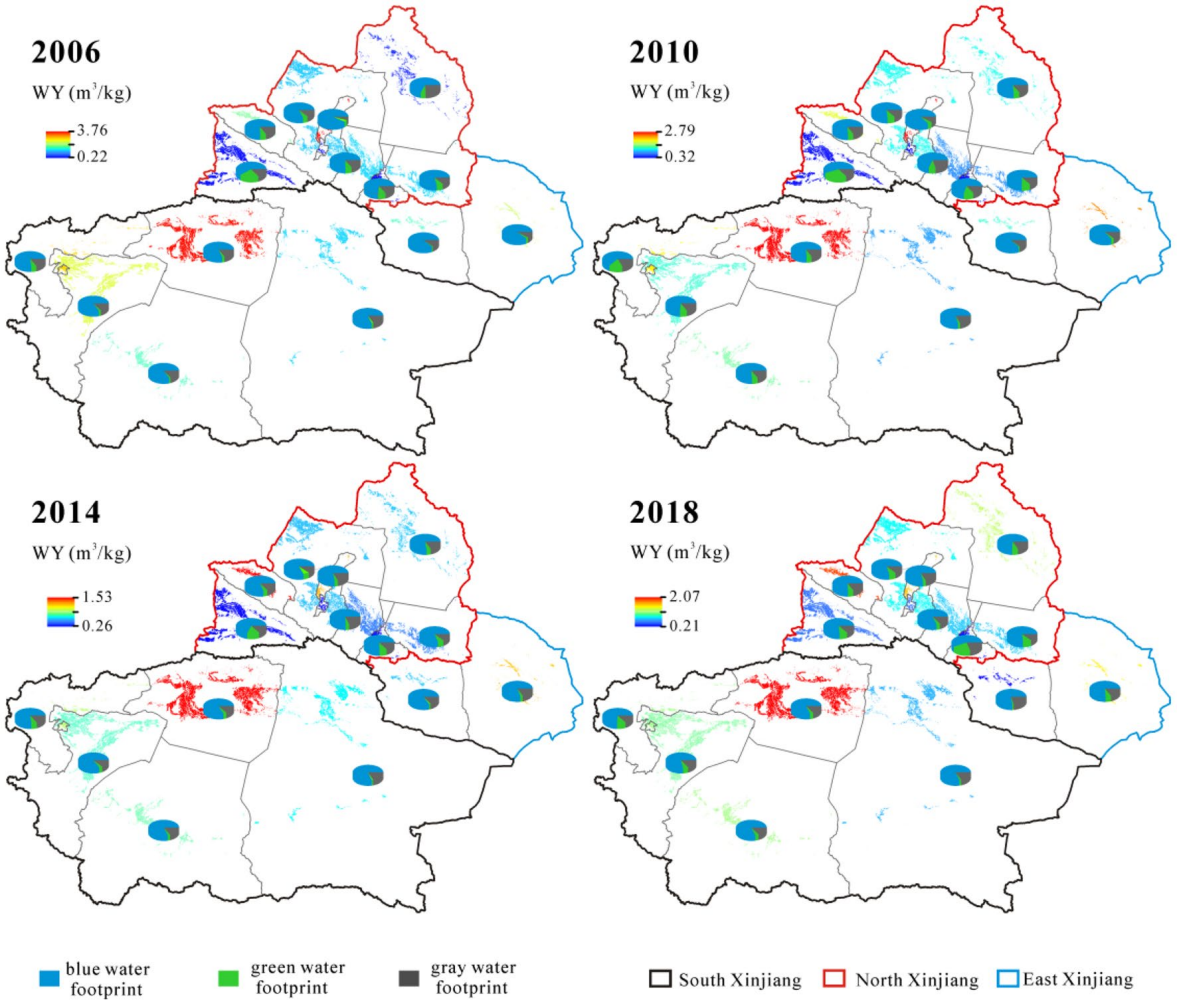
Spatial distribution and structure of water footprint per unit of yield (WY). Map is created in ArcGIS 10.1.

The WY has two trends in the study interval: an increased trend from 0.69 m^3^/kg in 2006 to 0.81 m^3^/kg in 2014 and then decreased to 0.80 m^3^/kg in 2018 (Fig. 6). In terms of WY_green_, WY_blue_ and WY_gray_, they all increased during 2006–2018. Specifically, WY_green_ increased from 0.05 to 0.06 m^3^/kg, WY_blue_ increased from 0.54 to 0.63 m^3^/kg, and WY_gray_ increased from 0.10 to 0.12 m^3^/kg. Being inconsistent with the changeable trends of WY_green_, WY_blue_ and WY_gray_, their ratios changed from 6.68 to 7.88%, from 78.77 to 76.31%, and from 14.55 to 15.81%, respectively. In north Xinjiang, the WY increased from 0.53 to 0.73 m^3^/kg, of which the ratio of WY_green_ decreased from 11.51 to 9.10%, WY_blue_ increased from 73.98 to 77.06%, and WY_gray_ decreased from 14.51 to 13.83%. In south Xinjiang, the WY decreased from 0.91 to 0.88 m^3^/kg, of which the ratio of WY_green_ increased from 2.84 to 5.23%, WY_blue_ decreased from 81.52 to 79.57%, and that of WY_gray_ remained at around 15%. In east Xinjiang, the WY decreased from 0.81 to 0.70 m^3^/kg, of which the ratio of WY_green_ increased from 2.00 to 5.92%, WY_blue_ decreased from 87.21 to 76.26%, and WY_gray_ increased from 10.77 to 17.81%. In terms of prefectures (Fig. 6), WY of Shihezi was the largest (2.07 m^3^/kg), while that of Urumqi was the smallest (0.21 m^3^/kg) in 2018. During 2006–2018, the larger increases of WY were showed in Altai (from 0.39 to 0.89 m^3^/kg) and Shihezi (from 1.10 to 2.07 m^3^/kg), while the obviously decreased WY were presented in Karamay (from 3.76 to 1.09 m^3^/kg) and Turpan (from 0.73 to 0.41 m^3^/kg).

**Figure 7:**
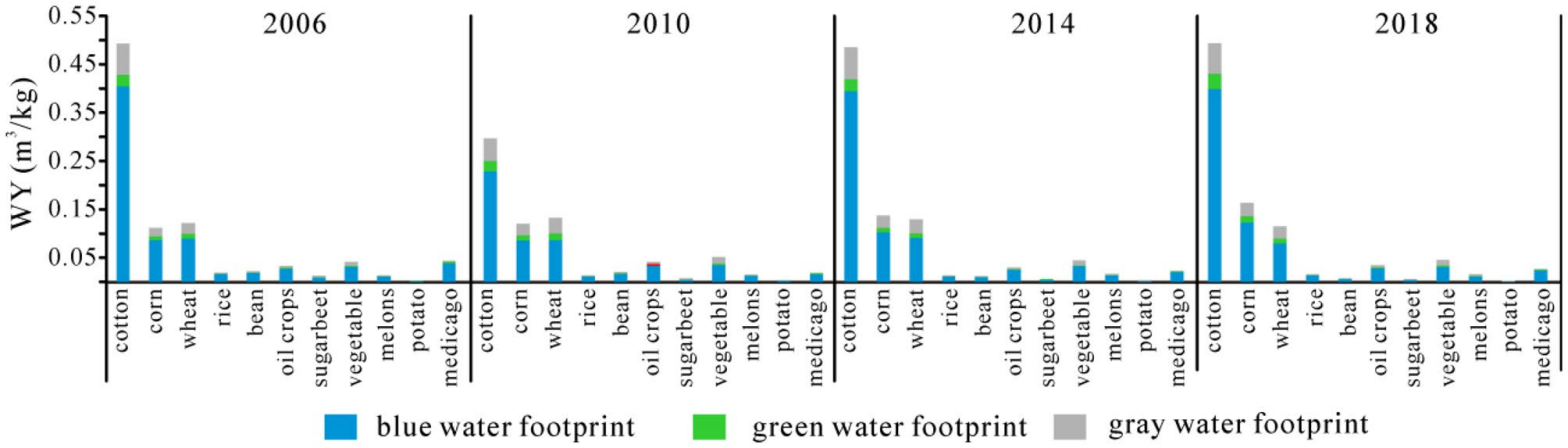
Water footprint per unit of yield (WY) in different crops.

In terms of crops, the averaged WY from high to low in turn were cotton (0.44 m^3^/kg), corn (0.13 m^3^/kg), wheat (0.12 m^3^/kg), vegetable (0.05 m^3^/kg), oil crops (0.04 m^3^/kg), medicago (0.03 m^3^/kg), rice (0.02 m^3^/kg), bean (0.02 m^3^/kg), melons (0.02 m^3^/kg), sugarbeet (0.01 m^3^/kg) and potato (0.002 m^3^/kg) (Fig. 7). In terms of the changing trends for crops, the largest decrease of 68.18% from 0.02 to 0.01 m^3^/kg was showed in soybean, while the smallest decrease of of 5.71% from 0.12 to 0.11 m^3^/kg was found in wheat. Corn has the largest increase from 0.11 to 0.16 m^3^/kg.

### Water footprint per output value (WV)

**Figure 8:**
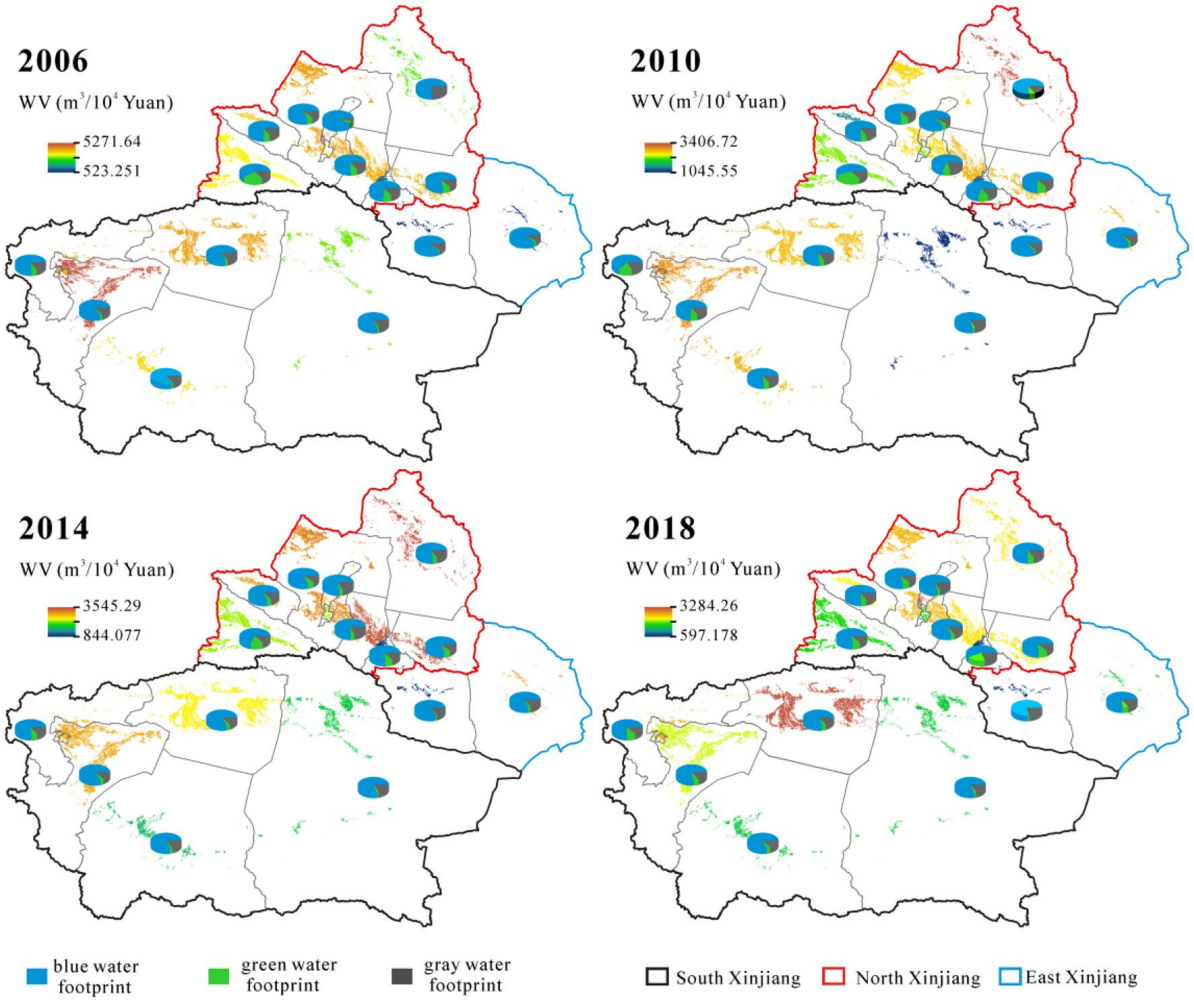
Spatial distribution and structure of water footprint per unit of GDP (WV). Map is created in ArcGIS 10.1.

The WV reduced from 4225.03 m^3^/10^4^ Yuan in 2006 to 2387.72 m^3^/10^4^ Yuan in 2018 (Fig. 8). The ratio of WV_blue_ dropped from 78.77 to 76.31%, while the ratios of WV_green_ and WV_gray_ increased from 6.68 and 14.55% to 7.88 and 15.81%, respectively. These changes are consistent with the trends of WY (Fig. 6). The similar trends of WV are found in south and north Xinjiang. Specifically, the WV reduced from 4302.50 m^3^/10^4^ Yuan to 2169.12 m^3^/10^4^ Yuan in south Xinjiang with a rate of 49.58%, and it reduced from 4031.39 m^3^/10^4^ Yuan to 2038.82 m^3^/10^4^ Yuan in north Xinjiang with a rate of 49.42%. Being converse with the trend of south and north Xinjiang, the WV increased from 761.29 m^3^/10^4^ Yuan to 1047.82 m^3^/10^4^ Yuan in east Xinjiang characterized by a rate of 37.64%. All prefectures (except Hami) have a decreasing trend of WV. The largest decline from 5271.64 m^3^/10^4^ Yuan to 2010.34 m^3^/10^4^ Yuan was observed in Shihezi. The second largest decline from 4908.22 to 2044.67 m^3^/10^4^ Yuan was in Kashgar.

Regarding the contribution rate in three regions (Fig. 8), the rate slightly decreased for WV_green_ from 9.46 to 8.42% and increased for WV_gray_ from 14.21 to 15.31% in north Xinjiang. The ratio of WV_blue_ remained stable at around 76%. In south Xinjiang, the ratio of WV_blue_ decreased from 80.74 to 78.73%. The ratio of WV_green_ increased from 3.58% in 2006 to 9.86% in 2010 and then decreased to 6.37% in 2018. The ratio of WY_gray_ decreased from 15.68 to 14.89%. In east Xinjiang, the ratios of WV_green_ and WV_gray_ increased from 1.53 and 6.12% to 11.68 and 17.64%, respectively. The ratio of WV_blue_ decreased from 86.99 to 76.23% during 2006–2018.

**Figure 9:**
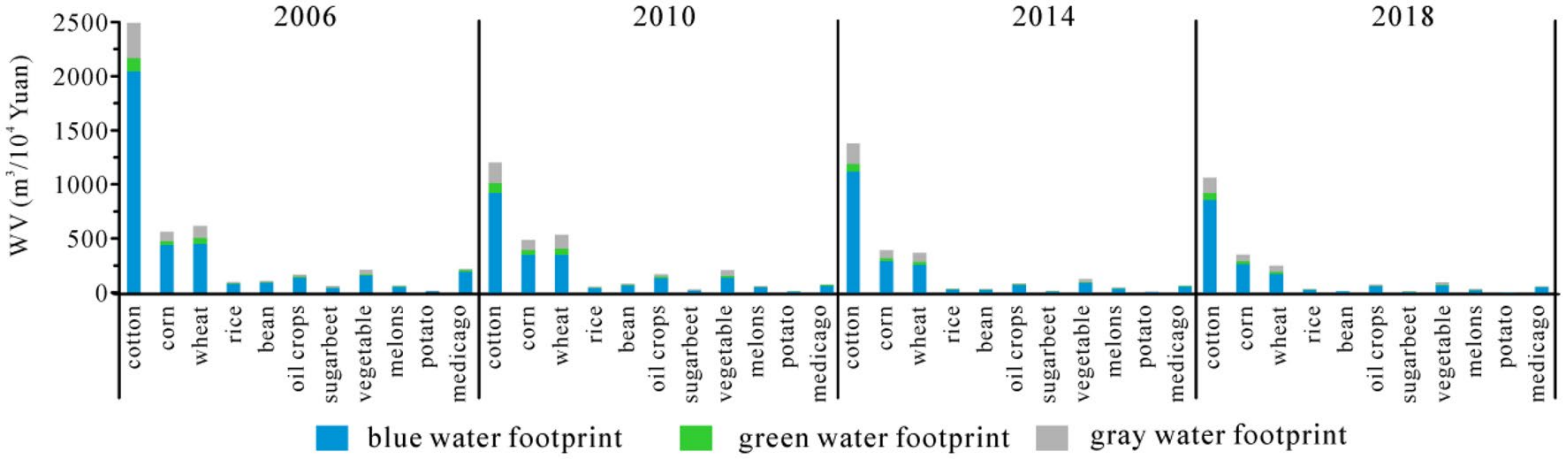
Water footprint per unit of GDP (WV) in different crops.

The averaged WV for crops were showed from high to low: cotton (1532.91 m^3^/10^4^ Yuan), corn (447.35 m^3^/10^4^ Yuan), wheat (440.76 m^3^/10^4^ Yuan), vegetable (160.04 m^3^/10^4^ Yuan), oil crops (123.32 m^3^/10^4^ Yuan), medicago (104.37 m^3^/10^4^ Yuan), bean (60.33 m^3^/10^4^ Yuan), rice (55.83 m^3^/10^4^ Yuan), melons (52.01 m^3^/10^4^ Yuan), sugarbeet (30.03 m^3^/10^4^ Yuan) and potato (10.56 m^3^/10^4^ Yuan) (Fig. 9). The WV of cotton had a tendency of decreasing, increasing and then decreasing. The WV of other crops decreased continuously. Being consistent with the ratios of WY_green_, WY_blue_ and WY_gray_, the ratio of WV_blue_ was the highest (mean 40.78%) in cotton, while the lowest (mean 0.25%) was found in potato.

## Discussion

### Water footprint and blue water footprint deficit

The main crop acreage in Xinjiang quickly amplified from 3.86 × 10^6^ ha in 2006 to 5.57 × 10^6^ ha in 2018, of which the yield of main crops increased from 3.19 × 10^10^ kg in 2006 to 4.66 × 10^10^ kg in 2018. It would inevitably result in a rise of WF from 22.75 to 44.16 billion m^3^ (Fig. 2). The rapid expansion of agricultural planting scale is the fundamental reason for the large increase of crops WF in Xinjiang^8,20,21^. The WF were showed from high to low in Xinjiang: WF_blue_ > WF_gray_ > WF_green_. It means that WF_blue_ is the most important water consumption and the role of WF_gray_ should be concerned in next researches because of WF_gray_ > WF_green_. In terms of the changeable rates, their increasing trend is also WF_blue_ (160.20 million m^3^/yr) > WF_gray_ (26.91 million m^3^/ yr) > WF_green_ (1.10 million m^3^/yr).

In terms of the increased rates of WF in three regions, they were significantly different: south Xinjiang (0.79 billion m^3^/yr) > north Xinjiang (0.82 billion m^3^/yr) > east Xinjiang (0.03 billion m^3^/yr) (Fig. 3). The WF in south Xinjiang (22.34 billion m^3^ in 2018) is consistently higher than that in north Xinjiang (20.61 billion m^3^ in 2018) in the studied interval, the gap between them was further shrinking from 2.11 billion m^3^ in 2006 to 1.73 billion m^3^ in 2018 due to its better agricultural development conditions, faster agricultural infrastructure construction and better large-scale operation foundation in north Xinjiang^16^. Correspondingly, the increased rate is WF_blue_ > WF_gray_ > WF_green_ in three regions. For WF_blue_ and WF_gray_, the rates are south Xinjiang > north Xinjiang > east Xinjiang. It means that the crop irrigation water consumption and fertilizer consumption in south Xinjiang were higher than those in north Xinjiang. For WF_green_, the rates are north Xinjiang > south Xinjiang > east Xinjiang. The increasing rates are consistent with significantly increasing precipitation in Xinjiang during 2006–2018^22^, which lead to higher effective precipitation (90–95%) in the conditions of the small amount of deep seepage and the majority surface-soil interception^8^. The expanded planting area also plays an significant role in an increase of WF_green_, which makes the area of crops can withstand precipitation increase and then makes the utilization of effective precipitation increase correspondingly^18,19^. In addition, the replacement of natural oases by many expansion of planting areas results in the conversion of ecological environmental water into artificial consumption in terms of water consumption^8^.

In Xinjiang, the BWF_d_ had an increased level from − 0.18 billion to 25.56 billion m^3^ during 2006–2018. The BWF_d_ of north Xinjiang (8.24 billion m^3^) and south Xinjiang (4.65 billion m^3^) were totally higher than that in east Xinjiang (0.38 billion m^3^). In terms of prefectures (Fig. 5), Aksu (2.23 billion m^3^), Bazhou (2.16 billion m^3^) and Kashgar (1.40 billion m^3^) experience the heaviest blue water deficit in south Xinjiang, while Bozhou (1.31 billion m^3^), Shihezi (2.90 billion m^3^), Tacheng (2.32 billion m^3^) and Changji (2.07 billion m^3^) experience the heaviest blue water deficit in north Xinjiang in 2018. This means these prefectures are the agricultural water-saving core area. Combined with the crop structures in prefectures, it is vital to optimize the planting structures of crops and to improve the irrigation coefficient and related management level with the purpose of slowing down the growth rate of WF in the blue-water-deficit prefectures of Xinjiang.

### Food productivity and economic benefits of water footprint

The issues of food safety have received increasing attention in the context of the explosive growth of population in Xinjiang and the availability of fresh water is the biggest challenge to food production. GDP is mainly affected by the fluctuating crop prices and the labor cost. From different angles (food productivity and economic productivity) of water footprint, it provides an alternative way for Xinjiang agriculture to save water.

As shown in Figs. 6, 7, 8, 9, three types were found in the relationship between WY and WV. Firstly, WY and WV mutual matched among different crops. In 2018, the maximum WY (0.49 m^3^/kg) and WV (2490.35 m^3^/10^4^ Yuan) are found in cotton, and the minimum WY (0.0030 m^3^/kg) and WV (15.30 m^3^/10^4^ Yuan) are showed in potato. The similar correspondences were also found among other crops. Secondly, WY and WV matched in different regions. In 2006, WY from large to small was 0.91 m^3^/kg in south Xinjiang, 0.81 m^3^/kg in east Xinjiang, and 0.53 m^3^/kg in north Xinjiang, while WV was 4302.50 m^3^/10^4^ Yuan in south Xinjiang, 4031.39 m^3^/10^4^ Yuan in north Xinjiang, 761.29 m^3^/10^4^ Yuan in east Xinjiang. The consistent correspondent relationships were found in other years. Thirdly, WY and WV mismatched in different prefectures. In 2018, the WV was largest in Shihezi (2.07 m^3^/kg), while the largest WV was in Aksu (3284.26 m^3^/10^4^ Yuan). This means water footprint food productivity in Aksu was lower than that in Shihezi, but the water footprint economic productivity was reversed. Two aspects should be proposed based on the above analysis: (1) food productivity is well corresponded with economic benefits among crops and three regions, which means WV or WY plays a equivalent role in government and farmers’ decisions about crop planting structures in Xinjiang; (2) different policies should be made from different perspectives (food productivity and economic benefits) in prefectures of Xinjiang, being consistent with the results of Zhangjiakou City^3^.

### Next works

Xinjiang WF experienced a continuous increasing trend during 2006- 2018. The rapid expansion of agricultural planting scale is the fundamental cause for a significant increase of WF for crops in Xinjiang. Under the condition of available water shortage in Xinjiang, we focus on a scientific view for the adjustment and transformation of crop structures to reasonably allocate water resources in Xinjiang. However, two following aspects should be done in future: (1) the spatial–temporal matching characteristics between water footprint and socioeconomic factors in each prefecture are needed to analyse using mathematical models (e.g., Gini coefficient and imbalance index^23^); (2) the specific planting area of crops are available via the remote sensing and the field investigation. Emphasizing comprehensive consideration of a variety of social and economic factors and detailed planting distribution of crops, we can provide a detailed plan to put forward suitable measures and policies to adjust the crop structure for sustainable agricultural development in prefectures of Xinjiang.

### Data sources and methods

#### Data sources

Total 66 meteorological stations for Xinjiang were collected from China Meteorological Administration (http://data.cma.cn/). The selected parameters include the maximum temperature, the minimum temperature, mean monthly temperature, mean monthly precipitation, wind speed, air pressure, relative humidity and sunlight duration. The land use/cover data were downloaded from National Earth System Science Data Center, National Science & Technology Infrastructure of China (http://www.geodata.cn). The NLCD maps (1 km) were produced by visual interpretation of Landsat Thematic Mapper (TM) images^24^. Socioeconomic data (crop types, planting areas, crops yields and regional GDP) were compiled from Xinjiang Statistics Yearbook (China). Irrigation water consumption for prefectures in Xinjiang were obtained from Xinjiang Water Resources Bulletin (2006, 2010, 2014 and 2018).

## Methods

### Experimental design

The water requirements of 11 crops in their growing period were firstly calculated using the CROPWAT 8.0 and then the temporal and spatial features of water footprint of crop production were estimated in 2006, 2010, 2014 and 2018. Combined with the irrigation water consumption, the blue water footprint deficits were calculated to reveal the situation of blue water. Finally, water footprint per unit of yield and water footprint per unit of GDP were estimated to depict water productivity from the perspective of food production and economic benefits in Xinjiang.

### Data processing

#### Water footprint (WF) for crop production

It consists of blue water footprint (WF_blue_), green water footprint (WF_green_) and gray water footprint (WF_gray_). The total WF of 11 major crops in Xinjiang is evaluated based on the calculation method of water footprint showed in^25^:

$${\text{WF}} = {\text{WF}}_{{{\text{blue}}}} + {\text{WF}}_{{{\text{green}}}} + {\text{WF}}_{{{\text{gray}}}}$$

where WF_blue_ is surface and ground water consumed (evaporated) by the production of a commodity; WF_green_ is the consumption of green water during the growing period of crops, green water is actually the total amount of rain evaporation. WF_gray_ is a product refers to the amount of fresh water required to assimilate contaminants according to existing environmental water quality standards.

#### Green water footprint (WF_green_) and blue water footprint (WF_blue_)

To calculate WF_green_ and WF_blue_, reference crop evapotranspiration (ET_0_) was calculated through meteorological elements and crop evapotranspiration (ET_c_) was calculated through crop regulation coefficient (K_c_)^26^. Crop evapotranspiration includes evaporation of soil surface and transpiration of crop. The specific formula is as follows^26^.

$${\text{ET}}_{{{\text{crop}}}} = {\text{ET}}_{{{\text{blue}}}} + {\text{ET}}_{{{\text{green}}}} = {\text{K}}_{{\text{c}}} \times {\text{ET}}_{0}$$

$${\text{WF}}_{{{\text{crop}}}} = 10 \times {\text{A}} \times \sum\limits_{d = 1}^{\lg P} {ET}_{crop}$$

In this equation, ET_crop_ is crop evapotranspiration (mm/day); ET_blue_ is the evapotranspiration of crop blue water; ET_green_ represents the evapotranspiration of green water; K_c_ is the crop regulation coefficient (dimensionless); ET_0_ is the reference crop evapotranspiration (mm/day); factor 10 is the conversion of the depth unit mm to the volume unit m^3^ of water; A is the crop planting area; $\sum\nolimits_{d = 1}^{\lg P} {{\text{ET}}}_{crop}$ is the total evapotranspiration in the growing period of crops from the sowing date (the first day) to the harvest date, lgP represents the number of days in the growing period.

ET_green_ was determined by comparing the potential evapotranspiration and effective precipitation (P_e_) during the growing period of crops, when ET_c_ > P_e_, ET_green_ = P_e_, ET_blue_ = ET_c_-P_e_; When ET_c_ < P_e_, ET_green_ = ET_c_, ET_blue_ = 0.

$${\text{WF}}_{{{\text{blue}}}} = 10 \times {\text{A}} \times \mathop \sum \limits_{{{\text{d}} = 1}}^{{\lg {\text{P}}}} {\text{ET}}_{{{\text{blue}}}}$$

$${\text{WF}}_{{{\text{green}}}} = 10 \times {\text{A}} \times \mathop \sum \limits_{{{\text{d}} = 1}}^{{\lg {\text{P}}}} {\text{ET}}_{{{\text{green}}}}$$

#### Gray water footprint (WF_gray_)

In this study, we adopts the groundwater quality standard (GB/T14848-93) and water quality standard for irrigation (GB5084-2005) in China, that is, nitrate (N) is less than 0.02 g/L, farmland irrigation water salinity in general should not be higher than 1.7 g/L. We selected C_max_ = 1.7 g/L, the ambient background concentration of nitrogen is assumed to be 0, and the specific calculation formula of gray water is as follows^27^:
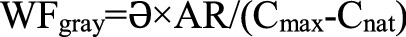


where WF_gray_ is the gray water footprint (m^3^); AR is the pure amount of nitrogen fertilizer, kg; Ə is the nitrogen leaching rate, %; C_max_ is the maximum environmental allowable concentration of nitrogen fertilizer (kg/m^3^); C_nat_ is the initial concentration of pollutants in water (kg/m^3^).

#### Blue water footprint deficit (BWF_d_)

Due to water shortage and imperfect water supply infrastructures, crops can’t always be fully irrigated in Xinjiang, WF_blue_ may not record the extent of blue water scarcity. In order to distinguish the actual blue water footprint consumption (WF_blue_′) from the crops requirement of blue water footprint, we used the blue water footprint deficit (BWF_d_) introduced by Ma et al.^3^ in this study, which refers to the difference between WF_blue_′ and WF_blue_.

$${\text{BWF}}_{{\text{d}}} = \frac{{\left( {{\text{WF}}_{{{\text{blue}}}} - {\text{WF}}_{{{\text{blue}}}}{}^{\prime } } \right)}}{{\upeta }}$$

When BWF_d_ < 0, it means a situation of blue water surplus. When BWF_d_ > 0, it means a situation of blue water shortage.

$${\text{WF}}_{{{\text{blue}}}}{}^{\prime } = {\text{W}}_{{\text{i}}} \times\upeta$$

where W_i_ refers to the irrigation water, ƞ is the effective utilization coefficient in each prefecture.

#### Water footprint per unit of yield (WY)

The WF per unit of yield (WY) is the WF divided by the crop yield (Y) and includes WF_blue_ per unit of yield (WY_blue_), WF_green_ per unit of yield (WY_green_) and WF_gray_ per unit of yield (WY_gray_)^3^.

$${\text{WY}}_{{{\text{blue}}}} = \frac{{{\text{WF}}_{{{\text{blue}}}}{}^{\prime } }}{{\text{Y}}}$$

$${\text{WY}}_{{{\text{green}}}} = \frac{{{\text{WF}}_{{{\text{green}}}} }}{{\text{Y}}}$$

$${\text{WY}}_{{{\text{gray}}}} = \frac{{{\text{WF}}_{{{\text{gray}}}} }}{{\text{Y}}}$$

$${\text{WY}} = {\text{WY}}_{{{\text{blue}}}} + {\text{WY}}_{{{\text{green}}}} + {\text{WY}}_{{{\text{gray}}}}$$

#### Water footprint per unit of GDP (WV)

The WF per unit of GDP (WV) is the WF divided by GDP^3^. It also includes three parts (WV_blue_, WV_green_ and WV_gray_), which reflects the economic benefits of WF.

$${\text{WV}}_{{{\text{blue}}}} = \frac{{{\text{WF}}_{{{\text{blue}}}}{}^{\prime } }}{{{\text{GDP}}}}$$

$${\text{WV}}_{{{\text{green}}}} = \frac{{{\text{WF}}_{{{\text{green}}}} }}{{{\text{GDP}}}}$$

$${\text{WV}}_{{{\text{gray}}}} = \frac{{{\text{WF}}_{{{\text{gray}}}} }}{{{\text{GDP}}}}$$

$${\text{WV}} = {\text{WV}}_{{{\text{blue}}}} + {\text{WV}}_{{{\text{green}}}} + {\text{WV}}_{{{\text{gray}}}}$$

#### Data analysis

According to the calculated results, we analyzed the temporal and spatial features of WF, BWF_d_, WY and WV in 2006, 2010, 2014 and 2018. The characters in these data among crops were also noted.

## Supplementary Information

Supplementary Information.

## References

[1] Cai Y (2016). Sustainable urban water resources management considering life-cycle environmental impacts of water utilization under uncertainty. Resour. Conserv. Recy..

[2] Meng B (2019). Water fluxes of Nenjiang River Basin with ecological network analysis: Conflict and coordination between agricultural development and wetland restoration. J. Clean. Prod..

[3] Ma W (2020). Spatiotemporal supply-demand characteristics and economic benefits of crop water footprint in the semi-arid region. Sci. Total Environ..

[4] Feike T (2017). Determinants of cotton farmers’ irrigation water management in arid Northwestern China. Agr. Water Manage..

[5] Li Y (2020). Estimation of regional irrigation water requirements and water balance in Xinjiang, China during 1995–2017. PeerJ.

[6] Liu X (2020). New challenges of food security in Northwest China: Water footprint and virtual water perspective. J. Clean. Prod..

[7] Zhao A (2016). Impacts of land use change and climate variability on green and blue water resources in the Weihe River Basin of northwest China. CATENA.

[8] Zhang P (2020). The spatiotemporal variations and the driving forces of agricultural water consumption in Xinjiang (1988–2015): Based on the statistical analysis of crop water footprint. J. Glaciol. Geocryol..

[9] Fovargue RE (2021). Spatial planning for water sustainability projects under climate uncertainty: Balancing human and environmental water needs. Environ. Res. Lett..

[10] Hoekstra, A.Y. Virtual water trade: Proceedings of the International Expert Meeting on Virtual Water Trade, Delft, The Netherlands, 12-13 December 2002, Value of Water Research Report Series No. 12, UNESCO-IHE, Delft, The Netherlands (2003).

[11] Mekonnen MM, Hoekstra AY (2011). The green, blue and grey water footprint of crops and derived crop products. Hydrol. Earth Syst. Sci..

[12] Chapagain AK (2006). The water footprint of cotton consumption: An assessment of the impact of worldwide consumption of cotton products on the water resources in the cotton producing countries. Ecol. Econ..

[13] Zhuo L (2016). The effect of inter-annual variability of consumption, production, trade and climate on crop-related green and blue water footprints and inter-regional virtual water trade: A study for China (1978–2008). Water Res..

[14] Cao X (2021). Water resources efficiency assessment in crop production from the perspective of water footprint. J. Clean. Prod..

[15] Cao X (2020). Hybrid analytical framework for regional agricultural water resource utilization and efficiency evaluation. Agric. Water Manag..

[16] Shen Y (2013). Estimation of regional irrigation water requirement and water supply risk in the arid region of Northwestern China 1989–2010. Agric. Water Manag..

[17] Wang F (2019). Assessment of the irrigation water requirement and water supply risk in the Tarim River Basin, Northwest China. Sustainability.

[18] Long A (2021). Understanding the spatial-temporal changes of oasis farmland in the Tarim River Basin from the perspective of agricultural water footprint. Water.

[19] Xuan JW (2014). Calculation and analysis on water footprint of main crops in Xinjiang. Agric. Res. Arid Areas.

[20] Xie P (2017). Precipitation and drought characteristics in Xinjiang during 1961–2015. Arid Land Geogr..

[21] Li YB (2020). Temporal-spatial variability of modern climate in the Altai Mountains during 1970–2015. PLoS ONE.

[22] Yao J (2016). Precipitation trend-elevation relationship in arid regions of the China. Glob. Planet. Chang..

[23] Ma W (2021). Spatiotemporal variations of agricultural water footprint and socioeconomic matching evaluation from the perspective of ecological function zone. Agric. Water Manag..

[24] Yang P (2018). Estimation of water consumption for ecosystems based on vegetation interfaces processes model: A case study of the Aksu River Basin, Northwest China. Sci. Total Environ..

[25] Hoekstra AY (2019). The Water Footprint of Modern Consumer Society.

[26] Allen, R.G., *et al.* Crop evapotranspiration-Guidelines for computing crop water requirements-FAO Irrigation and drainage paper, pp. 56 (1998).

[27] Zhang Y, Shi X (1998). Groundwater Hydrology.

