# Supplementary material for: Spatiotemporal variations of agricultural water footprint and its economic benefits in Xinjiang, northwestern China
Source: Sci Rep. 2021 Dec 13;11:23864. doi: 10.1038/s41598-021-03240-9 (PMC8668930; doi:10.1038/s41598-021-03240-9)
Supplement: Supplementary file 1 — Supplementary Information. [file 41598_2021_3240_MOESM1_ESM.doc]

**Supplementary Table S1** The planting area and yield of main crops in Xinjiang, China

| Crop type | 2006 | | 2010 | | 2014 | | 2018 | |
| --- | --- | --- | --- | --- | --- | --- | --- | --- |
|  | Area  (10^3^ ha) | Yield (10^4^ ton) | Area  (10^3^ ha) | Yield  (10^4^ ton) | Area  (10^3^ ha) | Yield  (10^4^ ton) | Area  (10^3^ ha) | Yield  (10^4^ ton) |
| Cotton | 1684.07 | 218.01 | 1445.32 | 247.90 | 2670.44 | 451 | 2491.3 | 511.1 |
| Corn | 496.43 | 386.80 | 742.74 | 421.61 | 960.43 | 641.09 | 1033.29 | 827.57 |
| Wheat | 740.11 | 400.31 | 1204.26 | 623.49 | 1240.86 | 642.27 | 1031.47 | 571.89 |
| Rice | 68 | 59.27 | 67.28 | 58.98 | 74.59 | 76.17 | 78.39 | 72.65 |
| Soybean | 96.14 | 25.01 | 127.72 | 28.32 | 85.18 | 21.67 | 44.51 | 13.96 |
| Oil crops | 151.73 | 76.50 | 273.4 | 102.50 | 220.52 | 118.3 | 224.13 | 57.25 |
| Sugarbeet | 94.42 | 32.82 | 75.27 | 66.63 | 62.88 | 59.33 | 57.26 | 67.81 |
| Vegetable | 186.82 | 555.53 | 303.61 | 486.97 | 310.59 | 471.94 | 273.26 | 424.73 |
| Melons | 74.02 | 958.04 | 122.79 | 1734.40 | 144.37 | 1819.79 | 111.35 | 1430.57 |
| Potato | 22.79 | 237.28 | 38.33 | 435.00 | 33.58 | 608.26 | 16.58 | 438.85 |
| Medicago | 242.38 | 240.15 | 150.15 | 132.93 | 206.01 | 225.10 | 211.62 | 244.07 |
| **Total** | **3856.91** | **3189.73** | **4550.87** | **4338.72** | **6009.45** | **5134.92** | **5573.16** | **4660.45** |
